# Differential regulation of p53 function by the N-terminal ΔNp53 and Δ113p53 isoforms in zebrafish embryos

**DOI:** 10.1186/1471-213X-10-102

**Published:** 2010-10-07

**Authors:** William R Davidson, Csaba Kari, Qing Ren, Borbala Daroczi, Adam P Dicker, Ulrich Rodeck

**Affiliations:** 1 Department of Radiation Oncology, Thomas Jefferson University, Philadelphia, PA, USA; 2 Department of Dermatology, Thomas Jefferson University, Philadelphia, PA, USA

## Abstract

**Background:**

The p53 protein family coordinates stress responses of cells and organisms. Alternative promoter usage and/or splicing of p53 mRNA gives rise to at least nine mammalian p53 proteins with distinct N- and C-termini which are differentially expressed in normal and malignant cells. The human N-terminal p53 variants contain either the full-length (FL), or a truncated (ΔN/Δ40) or no transactivation domain (Δ133) altogether. The functional consequences of coexpression of the different p53 isoforms are poorly defined. Here we investigated functional aspects of the zebrafish ΔNp53 ortholog in the context of FLp53 and the zebrafish Δ133p53 ortholog (Δ113p53) coexpressed in the developing embryo.

**Results:**

We cloned the zebrafish ΔNp53 isoform and determined that ionizing radiation increased expression of steady-state *ΔNp53 *and *Δ113p53 *mRNA levels in zebrafish embryos. Ectopic ΔNp53 expression by mRNA injection caused hypoplasia and malformation of the head, eyes and somites, yet partially counteracted lethal effects caused by concomitant expression of FLp53. FLp53 expression was required for developmental aberrations caused by ΔNp53 and for ΔNp53-dependent expression of the cyclin-dependent kinase inhibitor 1A (CDKN1A, p21, Cip1, WAF1). Knockdown of p21 expression markedly reduced the severity of developmental malformations associated with ΔNp53 overexpression. By contrast, forced Δ113p53 expression had little effect on ΔNp53-dependent embryonal phenotypes. These functional attributes were shared between zebrafish and human ΔNp53 orthologs ectopically expressed in zebrafish embryos. All 3 zebrafish isoforms could be coimmunoprecipitated with each other after transfection into Saos2 cells.

**Conclusions:**

Both alternative N-terminal p53 isoforms were expressed in developing zebrafish in response to cell stress and antagonized lethal effects of FLp53 to different degrees. However, in contrast to Δ113p53, forced ΔNp53 expression itself led to developmental defects which depended, in part, on p21 transactivation. In contrast to FLp53, the developmental abnormalities caused by ΔNp53 were not counteracted by concomitant expression of Δ113p53.

## Background

The p53 tumor suppressor coordinates the response of cells to genotoxic insults and other forms of cell stress either by inducing cell cycle arrest and allowing time for DNA repair or by causing elimination of damaged cells through apoptosis. The central role of p53 in cell stress responses lends significance to recent reports that p53 exists as a family of at least 9 different isoforms with different N- and C-termini due to differential splicing and promoter usage [1]. Three human N-terminal p53 isoforms are known containing either the full-length transactivation domain (FL), or a truncated transactivation domain (ΔN/p47) or no transactivation domain altogether (Δ133). The functional importance of some of these isoforms in vertebrate cells and organisms has only recently come into focus. Specifically, transgenic overexpression of ΔNp53 in mice leads to premature aging associated with increased expression of the CDK inhibitor CDKN1/WAF1/p21 and reduced proliferation [2]. In addition, both alternative N-terminal p53 isoforms have been observed to be differentially expressed in human tumor cell lines [1,3-5]. Finally, the zebrafish ortholog of the human Δ133p53 isoform (Δ113) is induced by genotoxic stress, counteracts FLp53 function and protects zebrafish embryos against deleterious consequences of genotoxic stress [6]. Whereas ΔNp53 expression is induced in an MDM2-dependent fashion in human colon carcinoma cells [4], expression and functional attributes of ΔNp53 in stress responses at the level of the whole organism have yet to be reported.

The present study seeks to investigate functional attributes of ΔNp53 in developing zebrafish embryos. We cloned the zebrafish *ΔNp53 *ortholog and, like *Δ113/133p53*, found it to be induced at the mRNA level by ionizing radiation (IR). Ectopic expression of zebrafish ΔNp53(z) isoform modulated the functional consequences of FLp53 expression in concert with Δ133p53 in zebrafish embryos. Furthermore, human ΔNp53(h) phenocopied the effects of zebrafish ΔNp53(z) when overexpressed in zebrafish embryos.

## Results and Discussion

### Identification and expression of zebrafish ΔNp53

**Figure 1:**
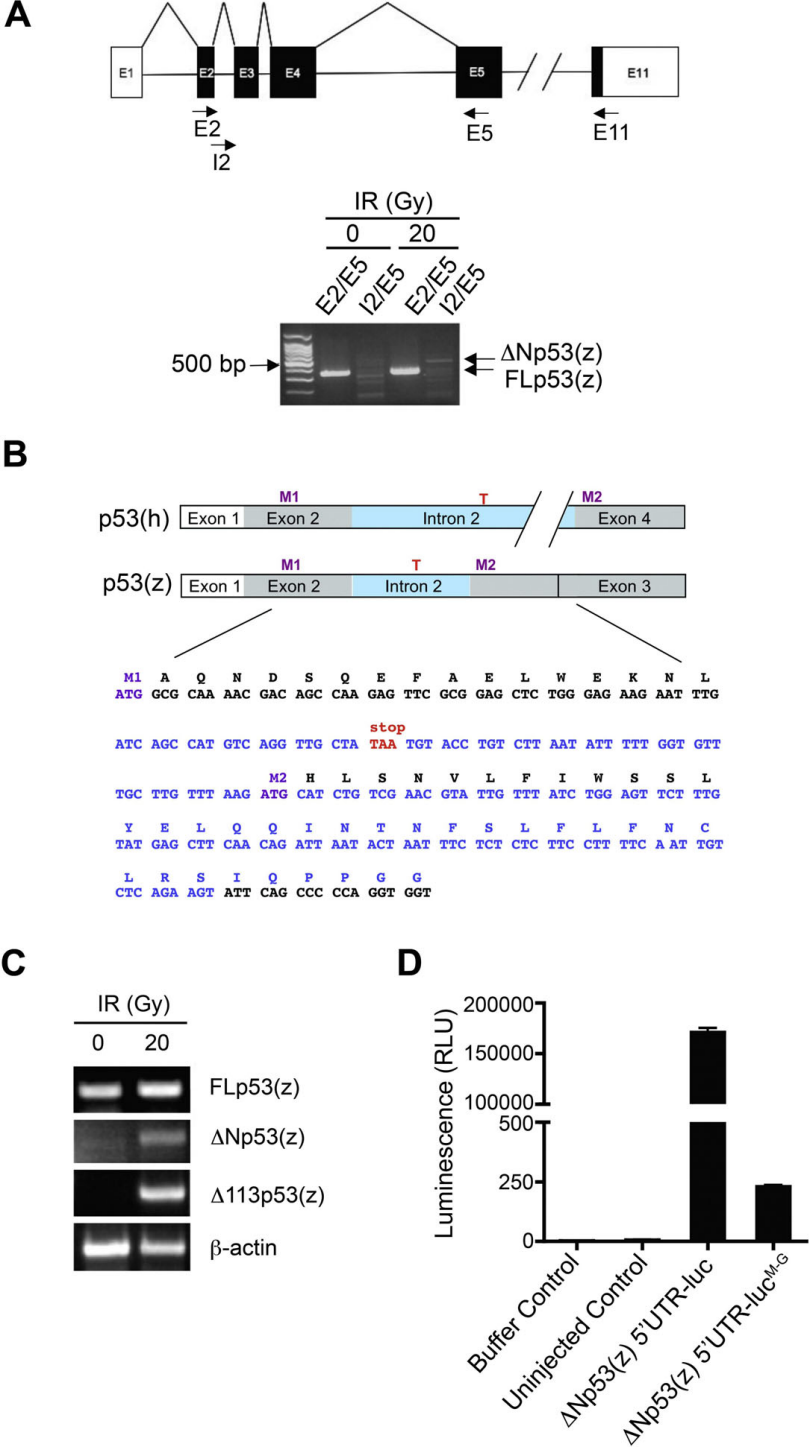
**Molecular attributes of zebrafish ΔNp53**. (A) RT-PCR analysis of zebrafish *ΔNp53*. The zebrafish *ΔNp53 *transcript was detected in cDNA preparations from irradiated embryos using the intron 2 forward primer (I2) and the exon 5 (E5) reverse primer. (B) Comparative view of human and zebrafish *ΔNp53 *sequences. In zebrafish, the alternatively spliced intron 2 (blue highlight) contains an in frame stop codon (T; red) and an alternate start codon (M2; purple) in frame with the *p53 *ORF of exon 3. (C) Semi-quantitative RT-PCR analysis of *p53 *isoform expression 6 h post irradiation of 24 hpf embryos. (D) Microinjection of 5'UTR-*ΔNp53 *M2-luciferase mRNA into zebrafish embryos. Strong expression of the luciferase reporter gene was achieved using this expression construct, which was abrogated by changing the putative M2 translation initiation site to glycine.

To amplify zebrafish *ΔNp53 *transcripts we designed a primer pair (I2/E5; Fig. 1; Table 1) that targets 5' sequences in intron 2 unique to a putative ΔNp53 and 3' sequences contained in exon 5 which are shared between all known p53 isoforms (Fig. 1A). An RT-PCR product of the expected size (456 bp) was identified in RNA preparations from 30 hpf embryos exposed to IR (20 Gy) at 24 hpf. Sequence determination of this amplification product revealed p53 sequences including intron 2 sequences (Fig. 1B). The predicted protein encoded by this transcript lacks part of the canonical transactivation domain but, unlike human ΔNp53, contains additional amino acids encoded by intron 2 which also contributes an alternative translation start site (M2; Fig. 1B). By semiquantitative RT-PCR, we confirmed that radiation exposure led to moderately increased steady-state *ΔNp53 *transcript levels in zebrafish embryos concomitant with increased *Δ113p53 *mRNA levels (Fig. 1C). These findings were consistent with prior work describing cell stress-induced expression of *Δ113p53 *mRNA in zebrafish [6]. Increased expression of ΔNp53 in human cell lines has similarly been observed in conditions of cell stress although this phenomenon has primarily been attributed to changes in protein abundance, rather than steady-state mRNA levels [7]. Although it is currently unknown how cell stress alters mRNA processing and splicing patterns, stress-associated alternative splicing has been observed for several mRNA species including the transcripts encoding MDM2 and MDM4 [8,9]. As expected, *FLp53 *transcript levels were comparable in irradiated and control embryos. Next, we verified that the putative alternative translation start codon M2 located in intron 2 of ΔNp53 was functional by microinjecting mRNA containing *p53 *exons 1-2 and intron 2 including M2 cloned upstream of and in-frame with the firefly luciferase gene. Expression of this construct in embryos resulted in high-level luciferase activity, which was not observed when the M2 methionine was changed to glycine (Fig. 1D). These results, in addition to the fact that exons 3 and 4 contain no in-frame translation initiation codons suggest that the M2 codon in intron 2 is the relevant alternative translation codon leading to ΔNp53 expression.

**Table 1:** Oligonucleotide sequences used for RT-PCR, RT-qPCR analyses and morpholino design

Primers for ΔNp53 PCR amplification (see Fig. 1A)		
Gene	Forward	Reverse

E2/E11	GCAAAACGACAGCCAAGAGT	ACGTCCACCACCATTTGAAC

I2/E5	CAGCCATGTCAGGTTGCTAT	CACCTTAATCAGAGTCGCTTC

		

**Primers for quantitative PCR (see Fig. 5)**		

Gene	Forward	Reverse

p21	GGAAAACATCCCGAAAACACC	TGTGATGTTGGTCTGTTTGCG

ΔNp53	CCTGATACACAACGCTCCTCT	TGATGTCCCAGCAAGAGCC

Δ113p53	ATTCTGTGTGACATTACAAGACCAGG	GTTTATTCAGGTCCGGTGAATACC

ELF1A1	CTTCTCAGGCTGACTGTGC	CCGCTAGCATTACCCTCC

		

**Primers for semiquantitative PCR (see Figs. 1C/2A)**		

Gene	Forward	Reverse

Δ113p53	TTTGGAGGGAGATGTTGGTC	GTTATCTCCATCCGGGGTTC

ΔNp53	TGCATCTGTCGAACGTATTG	GTTATCTCCATCCGGGGTTC

β-Actin	GATGTGGATCAGCAAGCAGGAGTA	CGAGAGTTTAGGTTGGTCGTTCGT

		

**Morpholino sequences**		

Gene	Sequence	

p53	5'GCGCCATTGCTTTGCAAGAATTG3'	

ΔNp53	5'AGGTACATTATAGCAACCTGACATG3'	

p21	5'TAATAAAGAGGTCTGACCTGTGATG3'	

standard control	5'CCTCTTACCTCAGTTACAATTTATA3'	

ΔNp53 mismatch control	5'AGcTAgATTATAGgAACCTcACtTG3'	

### Effects of forced ΔNp53 expression on zebrafish development

**Figure 2:**
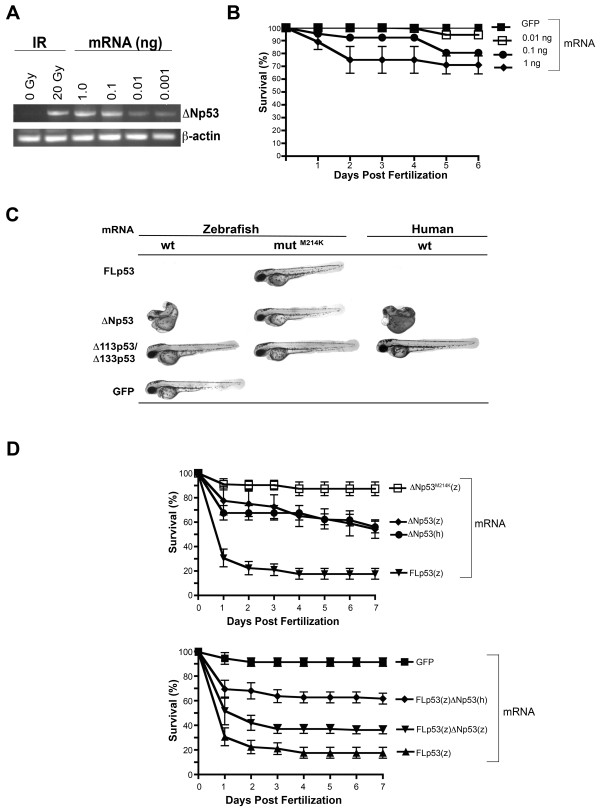
**Effects of ectopic expression of ΔNp53 on zebrafish embryos**. (A) Steady-state *ΔNp53 *message levels achieved by microinjection as compared to endogenous message levels in irradiated (20 Gy at 24 hpf) embryos; mRNA was isolated from 30 hpf embryos maintained in triplicate dishes of 60 embryos each and injected with 1-2000 pg of mRNA at the 2-4 cell stage. RT-PCR was performed as described in Material and Methods. (B) Embryo survival upon ectopic expression of *ΔNp53 *mRNA. Survival was defined as the presence of a heartbeat. (C) Representative examples of malformations caused by ectopic expression of p53 isoforms as evident at 48 hpf. Embryos were anesthetized with 0.003% tricaine, placed on 3% methylcellulose on a glass depression slide and examined using a fluorescence microscope (Leica MZ16FA) at 10× magnification. (D) Effects on embryo survival of 1 ng of either zebrafish or human ΔNp53 message either alone (upper panel) or in combination with zebrafish *FLp53 *mRNAs (lower panel). For control purposes, mRNAs encoding FLp53(z) and the functionally inactive M214K FLp53(z) mutant were included (upper panel). To ectopically express p53 isoforms, capped mRNAs were generated by cloning zebrafish cDNAs into pCS2+ and synthesized in vitro using the mMessage-mMachine-SP6 kit (Ambion).

**Figure 3:**
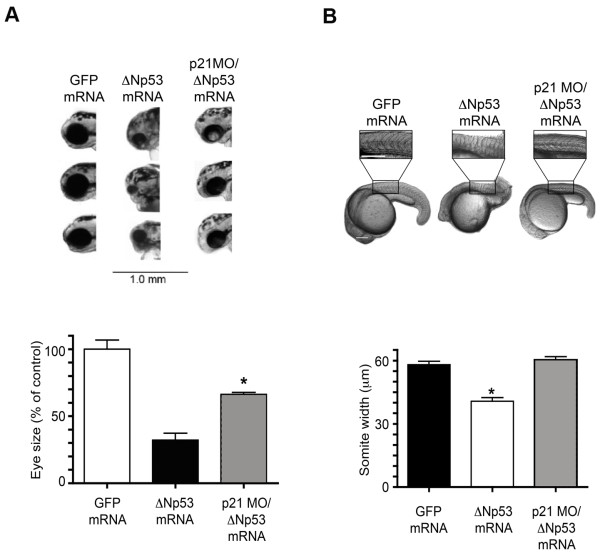
**Developmental malformations and hypoplasia caused by ectopic expression of zebrafish *ΔNp53 *mRNA**. (A) Representative images of the head region (upper panel) and quantification (lower panel) of eye size in embryos injected with either zebrafish *ΔNp53 *mRNA or GFP encoding control mRNA (1 ng/embryo). Eye size was determined by outlining the eye using ImageJ software (NIH) and calculating the eye area within the circumference. Results represent the mean ± SEM of 5 embryos. The asterisk denotes statistically significant difference (t-test, one tailed; p < 0.01). (B) Representative images of embryos and magnification of somites (upper panel) and quantification (lower panel) of somite size. Somite width was determined using ImageJ software (NIH). Results represent the mean of the width of five somites from three individual fish (mean ± SEM; n = 15). Asterices denote statistically significant differences (p < 0.001). The white bar in the GFP mRNA image represents 0.2 mm (10× magnification for all images). In addition to the effects of *ΔNp53 *mRNA the effects of coinjecting a p21-targeted MO are shown.

**Figure 4:**
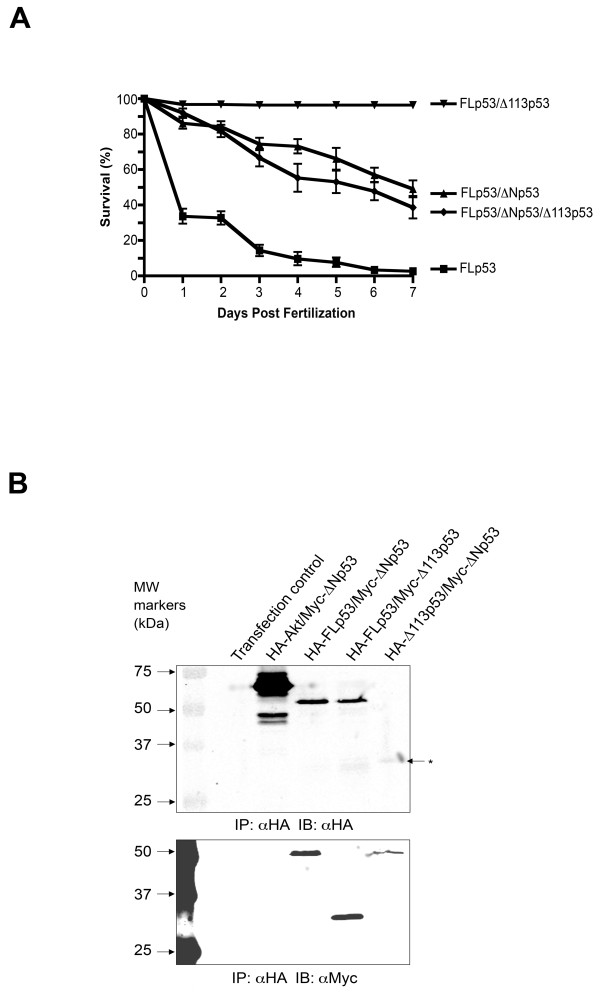
**Zebrafish survival in response to simultaneous ectopic expression of all three p53 isoforms in zebrafish embryos**. (A) Capped mRNAs encoding FLp53 (0.1 ng mRNA/embryo) alone or in combination with ΔNp53 (1 ng mRNA/embryo) or Δ113p53 (1 ng mRNA/embryo) or ΔNp53 (1 ng mRNA/embryo) together with Δ113p53 (1 ng mRNA/embryo) were injected into 1-4 cell embryos and survival was scored over seven days. Standard error was determined using triplicate dishes of 60 embryos each. Capped mRNA of green fluorescent protein (GFP; 2 ng RNA/embryo) was used as a negative control. (B) Molecular interaction of zebrafish p53 isoforms as determined by coimmunoprecipitation experiments using Saos-2 cells. All immunoprecipitations were performed using the HA Tag IP/Co-IP kit (Pierce, Rockford IL). The immunoprecipitates were subjected to SDS-PAGE and immunoblotted using antibodies recognizing the HA- and Myc-tags. The upper panel shows immunoblot detection of the HA-tag whereas the lower panel shows blots probed with antibodies detecting the Myc-tag. Experimental conditions were as follows: (1) Transfection control, (2) HA-Akt and Myc-ΔNp53 (3) HA-FLp53 and Myc-ΔNp53, (4) HA-FLp53 and Myc-Δ113p53, (5) HA-Δ113p53 and Myc-ΔNp53. The asterisk denotes the HA-Δ113p53 band. Expression of transgenes was validated by Western blot analysis of whole cell extracts (see Additional file 2).

We next determined the effects of ectopic ΔNp53 expression on zebrafish development using both human and zebrafish ΔNp53 orthologs. Injection of embryos with *ΔNp53 *mRNA of either species (1 ng/embryo) was calibrated to the endogenous *ΔNp53 *mRNA levels observed in 30 hpf embryos after IR (20 Gy at 24 hpf, Fig. 2A). Forced expression of either ΔNp53 ortholog induced lethality in approximately 30% of the embryos within 5-7 days (Fig. 2B/2D) associated with marked changes in morphology in the surviving embryos (Fig. 2C). Specifically, ΔNp53 overexpression induced hypoplastic and malformed heads, eyes and somites (Fig. 3). In addition, we observed moderately increased levels of apoptosis in *ΔNp53*-injected embryos as determined by acridine orange (AO) staining (Additional file 1). By comparison, ectopic expression of FLp53 alone (1 ng/embryo) led to embryonic lethality affecting the majority (> 80%) of early-stage (< 48 hpf) embryos (Fig. 2D) preceded by multiple morphological aberrations (not shown). Concurrent ectopic expression of mRNAs encoding ΔNp53 (either zebrafish or human) and FLp53(z) was associated with significant albeit not complete rescue of FLp53-associated lethality (Fig. 2D). Consistent with a recent report [6], the Δ113p53 isoform (1 ng/embryo) efficiently rescued lethal effects of forced FLp53 overexpression (Fig. 4A). As expected, injection of functionally impaired mutant p53 (M214K) isoforms [10] did not visibly affect zebrafish embryo viability or appearance (Fig. 2C, D). In aggregate, these results suggest that the relative abundance of the two isoforms in p53 tetramers is likely to affect the functional outcome of forced ΔNp53 expression.

### Combined effects of ectopic ΔNp53 and Δ113p53 isoform expression on FLp53-induced embryonal lethality

Previous work assessed the effects of transgenic overexpression of either ΔNp53 in mice [2] or Δ113p53 expression in zebrafish [6]. However, it is not known how simultaneous expression of the two alternative N-terminal p53 variants affects p53 responses of the whole organism. This is a relevant question because we observed coincident induction of ΔNp53 and Δ113p53 expression upon IR exposure of zebrafish embryos (Fig. 1C). To address this question directly we examined the consequences of ectopic expression of both alternative isoforms together with FLp53. To this end, we injected triple mRNA combinations encoding the different isoforms at different ratios ranging between 0.1 - 2 ng/embryo (Fig. 4A). These experiments confirmed that Δ113p53 was more efficient than ΔNp53 in rescuing FLp53-induced lethality. They further revealed that a combination of both alternative N-terminal isoforms does not add to the extent of rescue achievable with ΔNp53 alone (1 ng/ml). It is possible that morphological aberrations induced by ectopic ΔNp53 expression alone restrain the extent of rescue achievable by Δ113p53 expression. In contrast to ΔNp53 and as described previously [6], ectopic Δ113p53 expression, even at very high levels (2 ng/embryo) had no apparent effect on zebrafish morphology or development (not shown). Finally, ectopic expression of Δ113p53 (1 ng/embryo) did not affect the incidence of ΔNp53-dependent hypoplasia although the two isoforms could be coimmunoprecipitated (Fig. 4B and Additional file 2). At present, it is unclear why forced expression of Δ113p53 clearly counteracts the effects of FLp53 on zebrafish survival but not those of ΔNp53. It seems possible that the two alternative N-terminal isoforms in combination affect transcription of a subset of target genes that are deleterious to the developing embryo.

### The ΔNp53 isoform regulates p53 target gene expression in concert with FLp53

**Figure 5:**
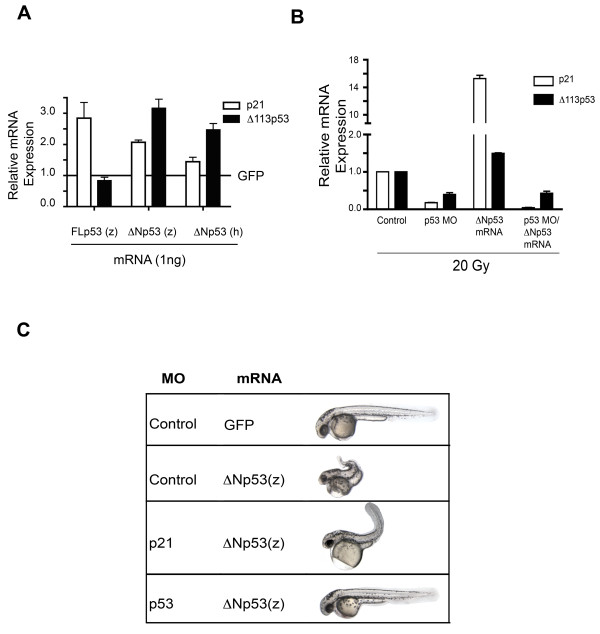
**Morphological alterations induced by ectopic ΔNp53 expression are partially rescued by knock-down of p21 expression and completely rescued by knock-down of FLp53**. (A) Quantitative RT-PCR analysis of steady-state *p21 *and *Δ113p53 *transcript levels at 30 hpf in unirradiated embryos microinjected with 1 ng/ml of mRNAs as indicated. (B) Quantitative RT-PCR analysis of steady-state *p21 *and *Δ113p53 *transcript levels in irradiated (20 Gy at 24 hpf) embryos harvested at 30 hpf. (C) Representative images of whole embryos (48 hpf) injected with 1 ng mRNA encoding *ΔNp53 *and MOs targeting either p21 [21] or FLp53 [11] or standard MO control (GeneTools). MO solutions (1 mM) were prepared in sterile water, diluted 3:1 (v:v) with Phenol Red, and 4 nL injected into 1-2 cell embryos.

Transgenic expression of ΔNp53 is associated with high-level expression of the cdk inhibitor CDKN1/p21/WAF1 expression in mice [2]. Similarly, and as determined by qRT-PCR we observed an increase in p21 message levels in zebrafish embryos injected with *ΔNp53 *mRNA (Fig. 5A) and this effect was even more pronounced in irradiated embryos (Fig. 5B). Interestingly, co-injection of a morpholino (MO) ablating FLp53 expression [11] prevented ΔNp53-dependent *p21 *mRNA induction indicating that ΔNp53 overexpression by itself is insufficient to upregulate p21 expression. To ascertain whether ΔNp53-dependent p21 expression was functionally involved in morphological alterations caused by ΔNp53 overexpression we ablated either p21 or FLp53 expression by coinjection of the respective MOs with *ΔNp53*(z) mRNA. Targeting p21 expression using a previously validated morpholino [12,13] markedly rescued hypoplasia of the eyes and somites caused by ectopic ΔNp53 expression (Fig. 5C and Fig. 3) and marginally reduced overall apoptosis (Additional file 3A). Consistent with the requirement of FLp53 for ΔNp53-dependent p21 induction, targeting FLp53 expression similarly alleviated hypoplasia in ΔNp53 overexpressing embryos and caused an even more robust reduction in apoptosis (Additional file 3B). We next determined whether Δ113p53 expression caused by IR in zebrafish embryos was similarly affected by ectopic ΔNp53 expression. We observed that forced ΔNp53 expression increased steady-state *Δ113p53 *transcripts and that, as in the case of *p21*, this effect was also contingent on the presence of FLp53 (Fig. 5A, B). Collectively, these results underscore that functional consequences of FLp53 overexpression are modified by coincident induction of either or both N-terminal p53 isoforms. In all experiments we used the p53α C-terminal isoforms, which form dimers and tetramers with each other [3,14] as confirmed for the zebrafish orthologs Δ113p53 [6] and ΔNp53 (Fig. 4B). To determine the functional importance of dimer/tetramer formation for these effects we used a ΔNp53 construct in which the C-terminal residues (302-374) that are homologous to the human oligomerization domain were deleted [15]. As expected, forced expression of C-terminally truncated ΔNp53 (Δ302-374) induced neither embryonal lethality nor the hypoplastic phenotype associated with forced expression of intact ΔNp53 α (Additional files 4A and 4B). Furthermore, C-terminally truncated ΔNp53 also did not affect p21 expression in irradiated embryos (Additional file 4C). In contrast and as expected, ablating endogenous ΔNp53 expression by a ΔNp53-specific MO in irradiated embryos moderately reduced *p21 *and Δ*113p53 *mRNA levels induced by IR albeit not to the extent achieved by FLp53-specific MO (Additional file 5). The MO used in this experiment targets untranslated sequences contained in intron2 upstream of the putative M2 transcription start site as indicated in Fig. 1. This MO is functional because it markedly (> 99%) reduced expression in zebrafish embryos of a reporter construct consisting of luciferase cloned downstream of the 5' UTR of *ΔNp53 *containing exons 1, 2 and intron 2 including the putative start site M2 (Additional file 5). Despite its effects on steady-state *p21 *and Δ*113p53 *transcripts, the ΔNp53-specific MO did not measurably affect overall survival of zebrafish embryos irradiated at 24 hpf. Effects of ΔNp53 knockdown on the radiation response of specific organ sites are currently under investigation.

## Conclusions

This study demonstrates that IR exposure of zebrafish embryos increased steady-state transcript levels of the two known alternative N-terminal p53 isoforms, Δ113p53 and ΔNp53. These isoforms shared the ability to counteract lethal effects of FLp53 expression in the developing fish. However, in contrast to Δ113p53, forced expression of ΔNp53 induced morphological aberrations itself, notably hypoplasia of the head and somites associated with a moderate degree of lethality. These effects of ΔNp53 were contingent on the presence of FLp53 and due, in part, to p21 induction. Furthermore, the developmental aberrations caused by forced expression of ΔNp53 limited the extent of rescue of FLp53-induced lethality by this isoform either alone or in combination with Δ113p53. Future work will address the relevance of ΔNp53 to the genotoxic stress response of specific tissues as induction of p53 targets occurs in a tissue-specific manner in mice [16] and ΔNp53 is expressed differentially in normal human tissues and malignancies [5].

## Methods

### Zebrafish Husbandry and Radiation Protocol

Zebrafish husbandry and embryo maintenance were performed according to standard procedures as published previously [17,18] and with approval by the IACUC at Thomas Jefferson University. Zebrafish were maintained at 28.5°C on a 14-h light/10-h dark cycle. Embryos were irradiated (20 Gy) at 24 hours post fertilization (hpf) using a 250 kVp X-ray machine (PanTak) at 50 cm source-to-skin distance with a 2-mm aluminum filter. Dosimetric calibration was performed before each experiment using a thimble ionization chamber with daily temperature and pressure correction.

### G-capped mRNA production

Generation of G-capped mRNA was performed using the mMessage-mMachine-sp6 kit (Ambion). Specifically, cDNA sequences were cloned into the pCS2+ vector containing an upstream sp6 promoter and a downstream SV40 polyA sequence. The vector was linearized using Sac II restriction enzyme (Promega) and 1 μg of linear plasmid was used as template for in vitro transcription according to the manufacturer's protocol. The mRNA was precipitated and diluted to 1 μg/μl in water prior to injection.

### 3 Prime Race, RT-PCR and RT-qPCR

The *ΔNp53 *transcript was cloned using the 3' RACE System (Invitrogen). First strand cDNA synthesis was performed using the Abridged Universal Amplification Primer (AUAP) and I2. A 1.7 kb amplification product consistent with *ΔNp53 *was isolated and purified for nested PCR using the primer pairs I2/E5 and I2/E11 yielding the expected 490 bp and 1.2 kb amplicons. The full-length 1.7 kb *ΔNp53 *cDNA was validated by direct sequencing.

For RT-PCR, RNA was prepared from 30-60 embryos using standard procedures. First strand cDNA synthesis for RT-PCR was done using Random Hexamers and AMV Reverse Transcriptase (Promega Madison). PCR was performed using Taq DNA Polymerase (Promega) and the GeneAMP PCR System 9700 (Applied Biosystems) with the following conditions: denaturation 95°C, 30 s; annealing 58°C, 30 s; extension 72°C, 1 min for 35 cycles. PCR products were analyzed in 1-2% agarose gels containing ethidium bromide. For primer sequences please refer to Table 1.

First strand cDNA synthesis for qRT-PCR was done using the High Capacity cDNA Reverse Transcription kit (Applied Biosystems). QRT-PCR was performed using the Power Syber Green PCR Master Mix and the ABI 7900 HT Sequence Detection System (Applied Biosystems) using the following conditions: denaturation 95°C, 30 s; annealing 55°C, 30 s; extension 72°C, 1 min for 40 cycles. For primer sequences please refer to Table 1. Relative mRNA expression levels were calculated using microinjection of control mRNA encoding green fluorescent protein (GFP).

### Reporter Gene Assay

The zebrafish *ΔNp53 *5'UTR sequence containing exons 1, 2 and intron 2 including the putative start site M2 and followed by an XhoI site was synthesized (DNA2.0 Menlo Park, CA) and inserted into the pCS2+ vector using BamHI-XhoI. The firefly luciferase gene was inserted into the XhoI/SnaBI site of pCS2+. ARCA-capped *ΔNp53*5'UTR-Luc mRNA was generated using mMessage-mMachine-sp6 kit (Ambion) and injected (1 ng/embryo) into 1-2 cell stage embryos. As a negative control the putative alternative start site (M2) was substituted with GGG encoding glycine by site-directed mutagenesis (Stratagene). Embryos (8 hpf) were assayed for luciferase activity using the Dual Luciferase Assay kit (Promega) and a luminometer (Turner Biosystems).

### Acridine orange staining of whole embryos

Embryos were stained with acridine orange as previously described with minor modifications [19]. To quantify apoptosis we used IMAGEJ software (NIH) to measure AO fluorescence intensity. Quantification of AO fluorescence intensity was determined by first converting the original images to greyscale. Using the Green stack (converted into greyscale) the embryo body excluding the yolk sac was selected. The image threshold was calibrated to control embryos to the point where controls exhibited the least pixel intensity within the outlined area. The ratio of white pixel area (representing green fluorescence intensity) to total area outlined was determined.

### Coimmunoprecipitation

Coimmunoprecipitation of zebrafish p53 isoforms was carried out after cotransfection into Saos-2 cells. Cotransfections were performed using pCS2+ expression vectors containing the following N-terminally tagged p53 isoform sequences in combination: 1) *HA-FLp53 *and *Myc-ΔNp53*, 2) *HA-FLp53 *and *Myc-*Δ*113p53*, 3) *HA-*Δ*113p53 *and *Myc-ΔNp53*. All transfections were done using the Fugene 6 Transfection Reagent (Roche, Germany) at a ratio of 3:1 (Fugene 6: DNA). Protein extracts were prepared 48 h post transfection and incubated with agarose beads conjugated with Anti-HA tag antibody. Immunoprecipitations were performed using the HA Tag IP/Co-IP kit (Pierce, Rockford IL). Interacting p53 isoforms were detected by immunoblot with Anti-Myc tag antibody. Coimmunoprecipitation of protein lysates from Saos-2 cells cotransfected with pCS2+ expression vectors encoding HA-Akt [20] and Myc-ΔNp53 was used as a negative control. Coimmunoprecipitation experiments were performed in triplicate and representative results are shown.

## Authors' contributions

WD cloned ΔNp53, performed RT-PCR and qRT-PCR experiments, prepared mRNA, microinjected zebrafish embryos and scored phenotypes, QR performed coimmunoprecipitation experiments, BD participated in microinjection and phenotypic characterization of fish and CK co-developed the cloning strategy and PCR assays. UR and APD conceived the study and wrote the manuscript. All authors have read and approved the final manuscript.

## Supplementary Material

Additional file 1 **Quantification of apoptosis in zebrafish embryo as determined by acridine orange staining**. Acridine orange staining was performed on 24 hpf embryos injected with zebrafish (z) or human (h) *ΔNp53 *mRNA (1 ng/embryo) as indicated. Statistically significant differences relative to control embryos injected with mRNA encoding GFP at p < 0.05 (one-tailed t-test) are indicated by an asterisk. A double asterisk indicates a statistically significant difference at p < 0.01. Representative images of 24 hpf embryos are shown in the right panels. Click here for file

Additional file 2 **Immunoblot analysis of protein extracts prior to immunoprecipitation**. To control for expression prior to coimmunoprecipitation immunoblot analyses were carried out on protein extracts of transfected Saos2 cells. Results shown are of immunoblot analysis of cell extracts using the HA- and Myc-tag antibodies. The upper panel shows the immunoblot using the anti-HA antibody. The lower panel shows the immunblot using the anti-Myc antibody. All proteins were expressed as expected. Note that the Myc antibody also detects endogenous Myc. Click here for file

Additional file 3 **Quantification of apoptosis associated with ectopic p53 isoform expression in zebrafish embryos**. Acridine orange staining was performed on 24 hpf embryos co-injected with zebrafish *ΔNp53 *mRNA and morpholinos (MOs) targeting either p21 (A) or FLp53 (B). Each column represents 5 embryos and images show representative embryos for each treatment group. Statistically significant differences (p < 0.05) relative to negative control (phenol red injected) are indicated by an asterisk. Representative images of 24 hpf embryos injected with mRNA/MO combinations are shown in the panels next to the bar graphs. Click here for file

Additional file 4 **Survival and morphological appearance of zebrafish embryo injected with C-terminally truncated ΔNp53(Δ302-374) mRNA**. (A) Triplicate dishes of 30 embryos each were injected with mRNA encoding GFP (control), Δ*Np53 *mRNA or Δ*Np53(*Δ*302-374) *mRNA lacking the dimerization domain. Survival was determined by the presence of a heartbeat was assessed over 7 days. (B) Upper panel: representative images of zebrafish embryos at 24 hpf. Lower panel: representative images of single embryos at 48 hpf. Bar (lower panel) = 0.5 mm. (C) Injection of Δ*Np53(*Δ*302-374) *mRNA did not affect steady-state p21 message levels (grey bar). This is in contrast to dimerization-competent Δ*Np53 *mRNA containing C-terminal sequences corresponding to the p53 α isoform used as a positive control (black bar). Click here for file

Additional file 5 **Characterization of Δ*Np53*-specific morpholino and effects on target gene expression**. (A) Efficacy of Δ*Np53 *knockdown by the morpholino directed against sequences in intron 2 of Δ*Np53*. The effect of Δ*Np53 *MO on a construct containing the 5' sequences of Δ*Np53 *driving luciferase expression and injected into zebrafish embryos at the 2-4 cell stage was measured and compared to the effects of a mismatch MO control (see Table 1). (B) Effect of Δ*Np53 *MO on *p21 *and Δ*113p53 *steady-state transcript levels as determined by RT-qPCR in irradiated embryos (IR (20 Gy) at 24 hpf, RNA isolation at 30 hpf). For comparison downregulation of target gene expression by *FLp53*-directed MO [11] are shown (grey bars). All results are shown were statistically different (p < 0.05; ANOVA-Bonferroni test) relative to mRNA expression levels of unirradiated embryos. Control mock-injected embryos (white bars) received 20 Gy at 24 hpf. Click here for file
