# Supplementary figures and images for: Differential regulation of p53 function by the N-terminal ΔNp53 and Δ113p53 isoforms in zebrafish embryos
Source: BMC Dev Biol. 2010 Oct 7;10:102. doi: 10.1186/1471-213X-10-102 (PMC2958959; doi:10.1186/1471-213X-10-102)

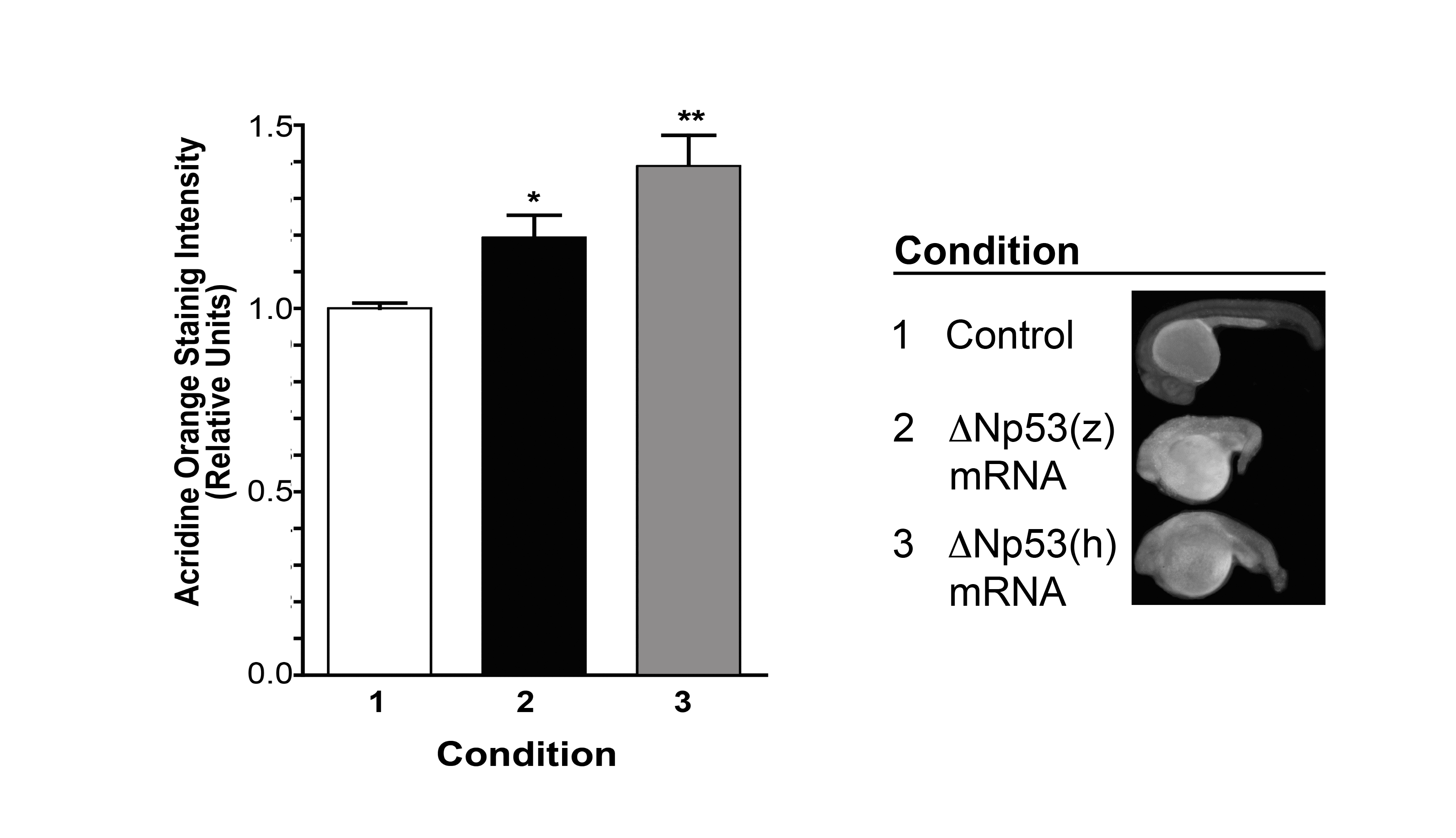

Supplement: Additional file 1 — Quantification of apoptosis in zebrafish embryo as determined by acridine orange staining. Acridine orange staining was performed on 24 hpf embryos injected with zebrafish (z) or human (h) ΔNp53 mRNA (1 ng/embryo) as indicated. Statistically significant differences relative to control embryos injected with mRNA encoding GFP at p < 0.05 (one-tailed t-test) are indicated by an asterisk. A double asterisk indicates a statistically significant difference at p < 0.01. Representative images of 24 hpf embryos are shown in the right panels. [file 1471-213X-10-102-S1.TIFF]

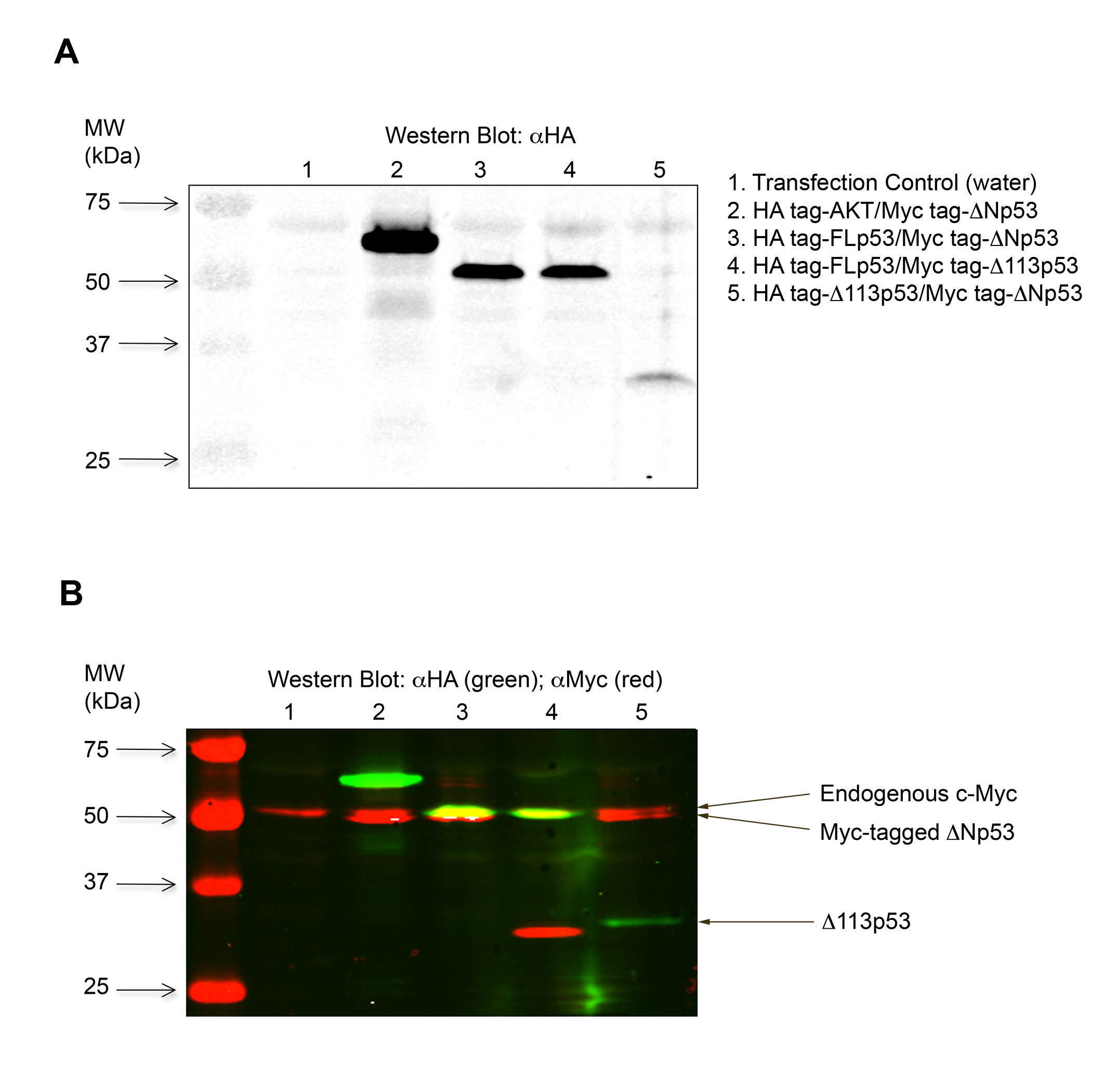

Supplement: Additional file 2 — Immunoblot analysis of protein extracts prior to immunoprecipitation. To control for expression prior to coimmunoprecipitation immunoblot analyses were carried out on protein extracts of transfected Saos2 cells. Results shown are of immunoblot analysis of cell extracts using the HA- and Myc-tag antibodies. The upper panel shows the immunoblot using the anti-HA antibody. The lower panel shows the immunblot using the anti-Myc antibody. All proteins were expressed as expected. Note that the Myc antibody also detects endogenous Myc. [file 1471-213X-10-102-S2.TIFF]

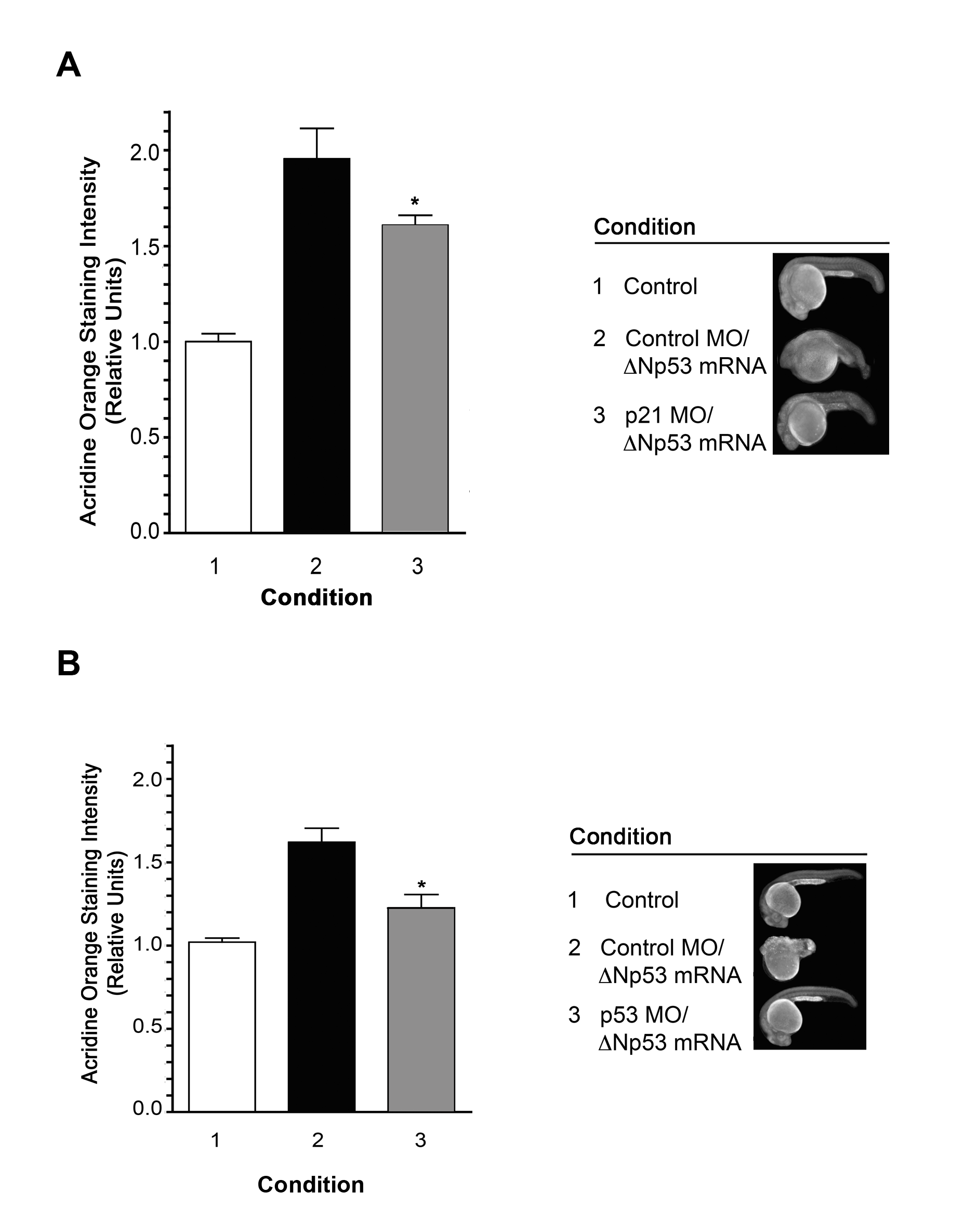

Supplement: Additional file 3 — Quantification of apoptosis associated with ectopic p53 isoform expression in zebrafish embryos. Acridine orange staining was performed on 24 hpf embryos co-injected with zebrafish ΔNp53 mRNA and morpholinos (MOs) targeting either p21 (A) or FLp53 (B). Each column represents 5 embryos and images show representative embryos for each treatment group. Statistically significant differences (p < 0.05) relative to negative control (phenol red injected) are indicated by an asterisk. Representative images of 24 hpf embryos injected with mRNA/MO combinations are shown in the panels next to the bar graphs. [file 1471-213X-10-102-S3.TIFF]

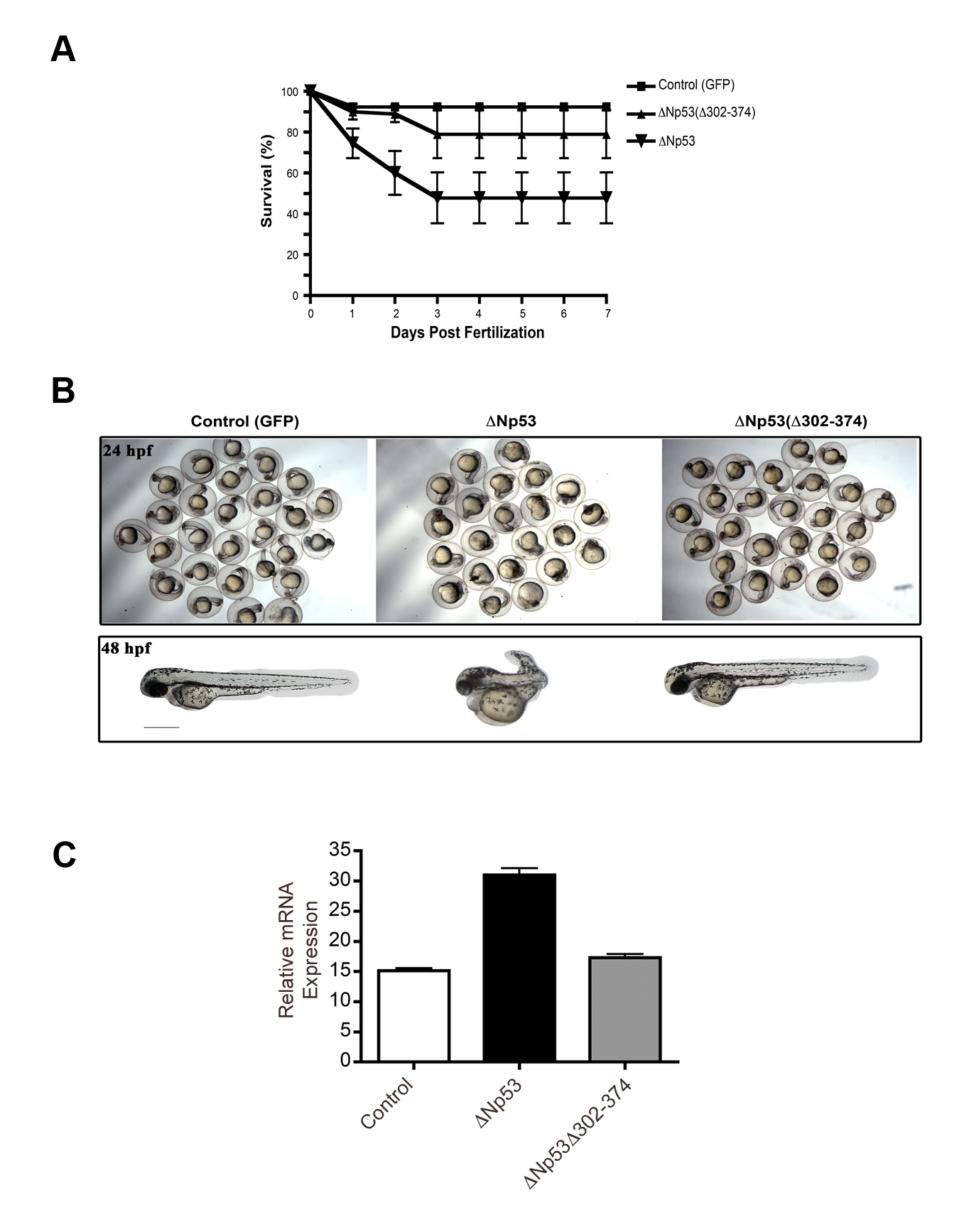

Supplement: Additional file 4 — Survival and morphological appearance of zebrafish embryo injected with C-terminally truncated ΔNp53(Δ302-374) mRNA. (A) Triplicate dishes of 30 embryos each were injected with mRNA encoding GFP (control), ΔNp53 mRNA or ΔNp53(Δ302-374) mRNA lacking the dimerization domain. Survival was determined by the presence of a heartbeat was assessed over 7 days. (B) Upper panel: representative images of zebrafish embryos at 24 hpf. Lower panel: representative images of single embryos at 48 hpf. Bar (lower panel) = 0.5 mm. (C) Injection of ΔNp53(Δ302-374) mRNA did not affect steady-state p21 message levels (grey bar). This is in contrast to dimerization-competent ΔNp53 mRNA containing C-terminal sequences corresponding to the p53 α isoform used as a positive control (black bar). [file 1471-213X-10-102-S4.TIFF]

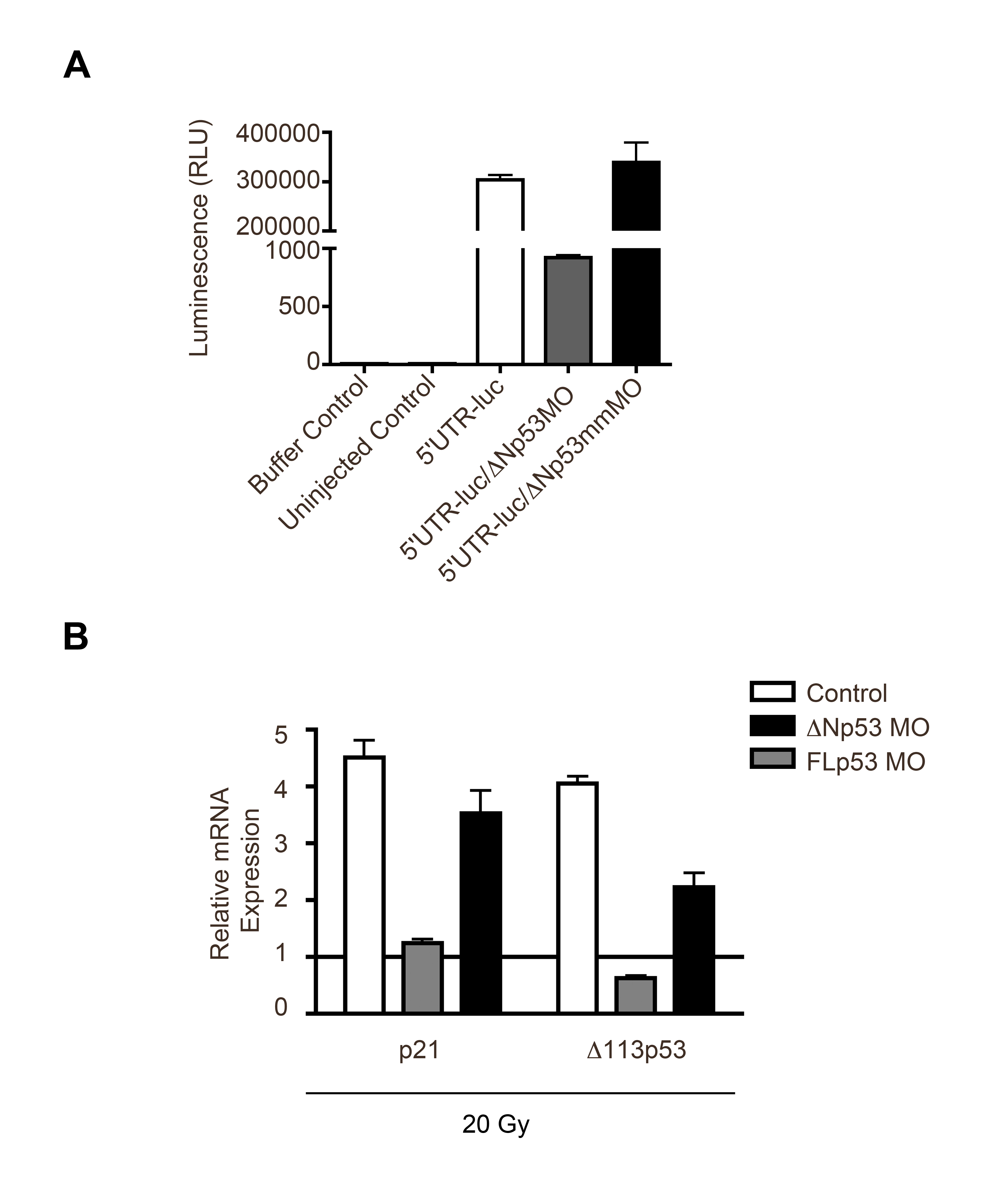

Supplement: Additional file 5 — Characterization of ΔNp53-specific morpholino and effects on target gene expression. (A) Efficacy of ΔNp53 knockdown by the morpholino directed against sequences in intron 2 of ΔNp53. The effect of ΔNp53 MO on a construct containing the 5' sequences of ΔNp53 driving luciferase expression and injected into zebrafish embryos at the 2-4 cell stage was measured and compared to the effects of a mismatch MO control (see Table 1). (B) Effect of ΔNp53 MO on p21 and Δ113p53 steady-state transcript levels as determined by RT-qPCR in irradiated embryos (IR (20 Gy) at 24 hpf, RNA isolation at 30 hpf). For comparison downregulation of target gene expression by FLp53-directed MO [11] are shown (grey bars). All results are shown were statistically different (p < 0.05; ANOVA-Bonferroni test) relative to mRNA expression levels of unirradiated embryos. Control mock-injected embryos (white bars) received 20 Gy at 24 hpf. [file 1471-213X-10-102-S5.TIFF]
